# The effect of mild sleep deprivation on diet and eating behaviour in children: protocol for the Daily Rest, Eating, and Activity Monitoring (DREAM) randomized cross-over trial

**DOI:** 10.1186/s12889-019-7628-x

**Published:** 2019-10-22

**Authors:** Aimee L. Ward, Barbara C. Galland, Jillian J. Haszard, Kim Meredith-Jones, Silke Morrison, Deborah R. McIntosh, Rosie Jackson, Dean W. Beebe, Louise Fangupo, Rosalina Richards, Lisa Te Morenga, Claire Smith, Dawn E. Elder, Rachael W. Taylor

**Affiliations:** 1 Department of Medicine, University of Otago, Dunedin, New Zealand; 2 Department of Women’s and Children’s Health, University of Otago, Dunedin, New Zealand; 3 Biostatistics Unit, University of Otago, Dunedin, New Zealand; 4 School of Health, Victoria University of Wellington, Wellington, New Zealand; 5 Department of Pediatrics, University of Cincinnati College of Medicine, Division of Behavioral Medicine and Clinical Psychology Cincinnati Children’s Hospital Medical Center, Ohio, USA; 6 Department of Paediatrics and Child Health, University of Otago, Wellington, New Zealand

**Keywords:** Obesity, child, sleep, dietary intake, eating behaviour, physical activity, sedentary behaviour

## Abstract

**Background:**

Although insufficient sleep has emerged as a strong, independent risk factor for obesity in children, the mechanisms by which insufficient sleep leads to weight gain are uncertain. Observational research suggests that being tired influences what children eat more than how active they are, but only experimental research can determine causality. Few experimental studies have been undertaken to determine how reductions in sleep duration might affect indices of energy balance in children including food choice, appetite regulation, and sedentary time. The primary aim of this study is to objectively determine whether mild sleep deprivation increases energy intake in the absence of hunger.

**Methods:**

The Daily, Rest, Eating, and Activity Monitoring (DREAM) study is a randomized controlled trial investigating how mild sleep deprivation influences eating behaviour and activity patterns in children using a counterbalanced, cross-over design. One hundred and ten children aged 8–12 years, with normal reported sleep duration of 8–11 h per night will undergo 2 weeks of sleep manipulation; seven nights of sleep restriction by going to bed 1 hr later than usual, and seven nights of sleep extension going to bed 1 hr earlier than usual, separated by a washout week. During each experimental week, 24-h movement behaviours (sleep, physical activity, sedentary behaviour) will be measured via actigraphy; dietary intake and context of eating by multiple 24-h recalls and wearable camera images; and eating behaviours via objective and subjective methods. At the end of each experimental week a feeding experiment will determine energy intake from eating in the absence of hunger. Differences between sleep conditions will be determined to estimate the effects of reducing sleep duration by 1–2 h per night.

**Discussion:**

Determining how insufficient sleep predisposes children to weight gain should provide much-needed information for improving interventions for the effective prevention of obesity, thereby decreasing long-term morbidity and healthcare burden.

**Trial registration:**

Australian New Zealand Clinical Trials Registry ACTRN12618001671257. Registered 10 October 2018.

## Background

Childhood obesity is a major issue worldwide, and New Zealand (NZ) is no exception; one in three children are overweight or obese with marked ethnic and socioeconomic inequities present [1]. Given established difficulties in treating childhood obesity [2, 3], early prevention is considered key [4]. However, changing diet and physical activity behaviours long-term has proven difficult among children as well as adults [2, 3], highlighting the need to examine alternative approaches to effective weight management. Interest is growing in the potential for sleep as a novel component of weight management [5, 6], given the extensive observational literature demonstrating short sleep duration as a strong, independent risk factor for obesity in children [7–10], and promising experimental evidence indicating that sleep interventions can substantially reduce the risk of obesity in early childhood [11–13]. However, it is currently not clear how insufficient sleep leads to weight gain [5, 14, 15], as the majority of research has been cross-sectional or cohort in nature limiting the ability to infer causality.

### How sleep affects energy intake

Observational literature suggests that not getting enough sleep influences what children eat, with shorter sleep duration associated with poorer quality diets [5, 16–19]. For example, using objective sleep measures, observational research has shown that sleep duration is negatively associated with energy density of the diet [5], independent of potential confounders such as screen time, physical activity, parental education level and ethnicity of the parents, suggesting that inadequate sleep is an independent risk factor for making poor nutritional choices in children [20]. Another study combined objective and subjective measures to report a one hour decrease in sleep duration over a 200 day period to be associated with higher intake of sugary beverages in children [17]. While most studies have measured dietary indices, very few studies actually measure energy intake; this is probably because the assessment of energy intake generally requires the use of diet recalls or food diary records, which pose a greater respondent and researcher burden. Of the limited research to date, one study showed short sleep duration to be associated with higher energy intake in infants [21], whereas no relationship was found in children [22].

Few experimental studies have manipulated sleep to determine the effects of measures of energy balance during growth, particularly in younger children. To date, only two trials have attempted to determine how dietary intake changes when sleep is experimentally restricted in children aged 12 and under, and these have both demonstrated that when sleep duration is reduced, energy intake increases [23, 24]. In these studies, reducing sleep by 2–3 h per night for up to five nights increased energy intake by 7–21%, a substantial amount on a daily basis, especially over time [23, 24]. However, these studies did not examine why energy intake differed - whether through a change in food choices, or because eating frequency or food quantity consumed increased. The reasons why sleep affects changes in energy intake in children need to be investigated in order to hone effective interventions.

### How sleep affects eating habits and behaviours

Sleep manipulation studies in children have tended to focus on adverse cognitive outcomes [25], but greater attention is now being paid to other aspects of sleep health [26], such as the association between decreased sleep duration and unhealthy lifestyle factors including dietary habits and activity levels [9, 10, 27]. A preference for energy-dense foods, irregular eating habits, and more external and emotional eating are all factors implicated in obesity [5, 16–19]. Despite huge international interest in sleep, experimental studies examining how being tired affects what, when and how school-aged children eat, are not common. Do children choose different foods? Does their appetite change? Do caregivers change what types of food they offer their tired, and possibly behaviourally challenging, child? These questions remain unanswered in experimental research, partly because of difficulties in measuring these important behaviours of importance, and partly because of the variety and quality of sleep measures used in studies, making quantification of studies in the field, and therefore providing causal statements about sleep and diet, problematic. Anecdotally, caregivers report that they parent differently when their child is tired, by being more conciliatory or by using treat foods as rewards. It has been established that when children are rewarded with food, this practice is associated with greater food intake [28]; however, no studies have systematically determined whether child-rearing behaviours around food and activity change when a child is sleep deprived.

Generally, relatively little is known about the context of eating, including what children might be doing at the same time (for example, socializing, watching television, or using screens) [29, 30]. However, some observational research suggests that a high degree of ‘food responsiveness’ (eating in response to factors other than hunger) at least partly mediates the relationship between sleep and weight in children [31]. Experimental studies in older children indicate that sleep deprivation was associated with choosing foods characterized by higher glycaemic index and glycaemic load with a trend toward more calories and carbohydrates [32, 33].

Although the measurement of the context of eating has previously been difficult [34], the advent of wearable technology, such as lapel cameras, offers a unique opportunity to examine what influences eating behaviour in children in a remarkably unobtrusive yet objective manner [35, 36]. Furthermore, given the well-established difficulties inherent in dietary assessment, wearable technology used in tandem with diet recall can enhance the accuracy of dietary reporting [37]. Applied here, such wearable technology could provide valuable complementary information to improve understanding of the effects of sleep deprivation on food intake itself, as well as context behind food intake, among children. For example, although screen time is known to increase dietary intake relative to non-screen behaviours in children, quantifying this effect has been difficult using questionnaires [38], and using camera images might allow for the quantification of the number of eating occasions per day where screens are present. Consumption of snacks, particularly less nutrient dense sweet and savoury snacks is already high in children during the evening hours [39]. Our research team has previously found that more than half of all meals captured on film during the evening were consumed whilst adolescents watched screens [40]. Additionally, social facilitation (a phenomenon when people eat more when they are in a bigger group), while evident in adults, requires more research in children [41, 42]. Using wearable cameras would allow for the examination of these questions passively in children, rather than in an artificial laboratory situation which may change behaviour.

### How sleep affects physical activity and sedentary time

Several studies of physical activity and sleep in children have shown a negative relationship between sleep duration and physical activity among children [9]. Shorter sleep duration has been associated with increased sedentary time, as the fatigue caused by short sleep increases lethargy and affects general mood [5, 43–45], and shorter sleep latency has been demonstrated in those children who participate in more physical activity during the day [46]. It makes sense to consider that sleep deprivation and the consequence of being tired might impact on physical activity in children; however, there is little experimental evidence that short sleep impacts on any component of energy expenditure, including physical activity [9, 47]. Methodological limitations may play a part in this [48]. As time spent asleep and time spent physically active both sit within a 24 h window, they are co-dependent so that if children sleep less, they have to do more of something else within those 24 h – e.g. spend more time in sedentary activities such as screen use, and/or more time being active. Whether or not reductions in sleep time are offset by increases in sedentary time or physical activity (measured via actigraphy, and compared by baseline versus intervention) is something that has not yet been investigated in children [49]. Only appropriate compositional analyses that account for the closed nature of the 24 h window and the co-dependence of these activities can determine the true effect of substituting one activity for another [48, 50]. National physical activity guidelines have recently promoted the importance of measuring all activity behaviours across the full 24-h day, that is, sleep, sedentary time, and physical activity [51, 52]. As such, collecting, analysing, and interpreting 24-h movement data becomes more important, but is complicated and requires appropriate statistical techniques that account for this compositional data [48].

Together, these findings suggest viable links between sleep and diet, but experimental studies are required to determine causality. The Daily Rest, Eating and Activity Monitoring (DREAM) study will assess the effect of mild sleep deprivation on eating behaviour in children aged 8–12 years, using both objective and subjective measurements. Use of a cross-over design reduces confounders, as each participant receives the same number of experimental manipulations over the same amount of time. Using technology in tandem with 24-h diet recalls will provide unique information on the environmental context of eating under different sleep conditions. By using well-established methods combined with novel approaches to improve understanding of how not getting enough sleep adversely affects health in children, we can potentially take the next steps in offering new pathways for decreasing the risk of obesity.

### Aims and objectives

The primary aim of the DREAM study is to determine whether mild sleep deprivation increases energy intake in the absence of hunger (measured objectively). Secondary aims will determine whether mild sleep deprivation: 1) influences the amount and types of foods eaten, 2) alters the context of eating (e.g. where, when, who with, what else is happening), 3) changes other indices of eating behaviour (e.g. emotional over-eating), 4) changes children’s preference for different food types, 5) changes how caregivers care for their child with regards to snacking and meals, and 6) influences time spent in sedentary or physical activity.

We hypothesize that, when compared to being well-rested, children whose sleep is restricted by one to 2 hrs per night will be less able to regulate their appetite, and will therefore eat more food, particularly at night, will eat for reasons other than hunger more often, will rate treat foods as more appealing, and will engage in more sedentary behaviour.

## Methods

The DREAM study is a randomized cross-over trial testing how sleep deprivation influences eating behaviour and activity patterns in children. We will manipulate the overnight sleep duration of healthy school-aged children aged 8–12 years who exhibit no sleep disturbances, in order to assess whether a mild level of sleep deprivation that is common in children affects what, when and how they eat. Using actigraphy across the full 24-h day to measure sleep, sedentary behaviour, and time spent in light, moderate and vigorous activity [43, 49], combined with wearable cameras to provide a passive, but highly informative way of “seeing” first-hand how children spend their time [35, 36], will provide novel data to determine whether sleep deprivation influences patterns of activity and eating behaviours. All children will undergo two experimental sleep conditions, sleep restriction and sleep extension.

### Study design and setting

**Fig. 1:**
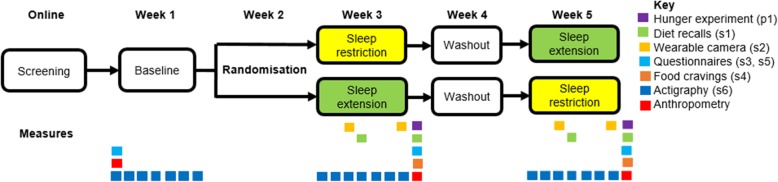
Study design

The DREAM study involves a five-week protocol including a baseline week to establish usual sleep patterns, followed by 1 week of sleep extension and 1 week of sleep restriction, with 1 week of no intervention in between. This design is illustrated in Fig. 1 and detailed in text. The study takes place in Dunedin, New Zealand.

Benefits of the cross-over design include a reduction in the influence of confounders as each child acts as their own control. The week between weeks 3 and 5 with no intervention should eliminate any carry-over effects from one condition to the other [53]. Period effects (that their first visit is different to their second, regardless of condition due to learning effects, or comfort levels at the study, etc.) will be accounted for by randomising to order and including this in the model. The difference between these two conditions should produce a reduction in “usual” sleep time of 1–2 h per night for each child, considered to be a level of mild sleep deprivation common in children [54]. Others report high compliance (95% retention) with similar protocols in children [55, 56],

We have chosen to use a combination of sleep restriction and extension (1 hour each for seven consecutive nights) rather than comparing 2 h of restriction against usual sleep (e.g. baseline against a week of restriction) as pilot work indicated this was more feasible and practical for families. As this is a cross-over trial and children are only compared with themselves, controlling for season is not necessary; however, to remove the potential modifying effect of school holidays, all data collection will be conducted during the school term. In this way, changing bed time while maintaining wake time reflects the real world where wake times remain relatively constant in children [57]. Participants will also undergo two 24-h diet recall sessions during each experimental week. As participants will commence the study on different days of the week, all days of the week will be represented. Wearable cameras will aid researchers in validating the diet recalls. At the end of each experimental week, participants will visit the clinic for a feeding study to assess eating in the absence of hunger. Finally, families will be invited to attend a qualitative exit interview after the study ends, by a researcher with mixed methods experience that has had no previous contact with the participant or caregiver. This qualitative interview will be open-ended and serve as an explanatory tool to compliment the study. All interviews will be audio recorded and professionally transcribed, and analysed thematically. Tables 1 and 2 describe DREAM study events and event locations.

**Table 1:** Timing of DREAM activities and study periods

Study activities	Study period			
	*Week 0*	*Week 1*	*Week 3*	*Week 5*
Eligibility screen (SDSC) [58]	X			
Informed consent		X		
Group allocation		X		
Interventions				
Sleep restriction/sleep extension			X	X
Sleep extension/sleep restriction			X	X
Outcome assessments:				
Child				
Actigraphy		X	X	X
Height		X		
Weight		X	X	X
EAH (Feeding experiment)			X	X
*Questionnaires:*				
Food cravings			X	X
Kidscreen [61]			X	X
PROMIS sleep disturbance [62]			X	X
PROMIS sleep impairment [62]			X	X
*Wearable camera			X	X
Primary caregiver				
*Questionnaires:*				
Demographics		X		
Child food allergy and preferences		X		
Child Sleep Hygiene [63]		X		
PROMIS sleep disturbance proxy [62]			X	X
PROMIS sleep impairment proxy [62]			X	X
Child eating behavior (CEBQ) [64]			X	X
Screen time			X	X
Snacks/meals			X	X
Child/caregiver dyad				
*Diet recall (in person) [65]			X	X

* = these measures are used twice in Weeks 3 and 5

**Table 2:** Location and description of DREAM study appointments

Study appointment number and descriptor	Location	Purpose of appointment
Appointment 1 – Baseline	Participant’s home	Collection of baseline data
Appointment 2 – DREAM week 1	Participant’s home	Set up for first experimental week
Appointment 3 – Diet recall	Participant’s home	1st diet recall; photo review
Appointment 4 – Feeding experiment 1	Metabolic kitchen (clinic)	2nd diet recall; feeding experiment; photo review
Appointment 5 – DREAM week 2	Participant’s home	Set up for second experimental week
Appointment 6 – Diet recall	Participant’s home	3rd diet recall; photo review
Appointment 7 – Feeding experiment 2	Metabolic kitchen (clinic)	4th diet recall; feeding experiment; photo review
Appointment 8 – Follow up interview	Participant’s home	Qualitative interview

### Participants and recruitment

We will recruit children aged 8 to 12 years through community networks and Facebook advertising. We will aim to recruit a sample of children that is broadly representative of New Zealand children to increase the generalisability of findings (e.g. 50% female, 30% overweight/obese, 60% European, 21% Māori, 11% Pacific, 11% Asian). Interested families will complete an online questionnaire assessing their child’s eligibility as follows: 8–12 years of age; reported time in bed of 8–11 h per night; no underlying medical condition (e.g. healthy children); and a score of 39 or less on the Sleep Disturbance Scale for Children [58]. Participants will be excluded outside of this age group, if they have a medical condition or take medication that affects sleep or eating behaviour, or sleep disturbances are indicated. Only children with reported time in bed (time between lights out and waking in the morning) of 8–11 h a night will be eligible to ensure that any sleep extension or restriction does not place them in the “not recommended” category for sleep duration according to international guidelines (< 7 or > 12 h) [59]. There should be little concern over potential safety aspects as our eligibility criteria ensure that children should not go below the minimum recommended sleep duration. Further support for this conclusion is provided by studies showing that any effect of sleep restriction on cognition, specifically vigilance, in children is surprisingly small [60],with no long-lasting effects on cognition because recovery from restricted sleep occurs quickly; thus the one week between intervention exposures is considered sufficient to eliminate effects of prior sleep deprivation [23, 66].

Primary caregivers (parents or legal guardians) of potentially eligible participants will access the screening questionnaire, the Sleep Disturbance Scale for Children (SDSC) [58], via the University of Otago Child and Teen Sleep Research Group on Facebook (see Table 1 for the timing of all study assessments). The well-validated SDSC will identify for exclusion those children who have potential sleep problems such as sleep-walking, restless legs, sleep disordered breathing, problems with initiating and maintaining sleep, and excessive sleepiness (score of ≤39 will be eligible). Total SDSC scores will be reviewed by a Paediatric Sleep Physician to make the final determination in regard to eligibility. Caregivers of eligible applicants will be contacted by a researcher to schedule study appointments. Caregivers of applicants who are deemed ineligible due to their SDSC score will be contacted via text or email with feedback from the Paediatric Sleep Physician review as to why their child was not considered eligible. This feedback will include advice on whether they should consult their Primary Care Physician in regard to their child’s sleep health. Upon completion of the study, participants will receive a $100 voucher, and their primary caregiver will receive a $50 voucher. Participants can withdraw from the study at any time.

### Study administration

Study data will be collected and managed using Research Electronic Data Capture (REDCap) tools hosted at the University of Otago [67]. REDCap is a secure, web-based application designed to support data capture for research studies, providing an intuitive interface for validated data entry, audit trails for tracking data manipulation and export procedures, automated export procedures for seamless data downloads to common statistical packages, and procedures for importing data from external sources. Practically speaking, REDCap allows for the tracking of participants, the dissemination of surveys, the managing of appointments, online questionnaire entry, and all other data entry in one place.

### Baseline and randomisation

Eligible participants will be seen at a 45–60 min baseline appointment in their home (week 1, Table 1). Primary caregivers will provide written consent for minors at this time, as well as complete online questionnaires assessing demographics (child date of birth, date of survey, household address to calculate household deprivation, ethnicity, family structure), child food preferences and allergies (to plan for the feeding experiment in weeks 3 and 5), and the Children’s Sleep Hygiene Survey [63]. Consent will also be gained from each child participant. Height (Wedderburn Portable Height Rod, WS-HRP) and weight (Tanita electronic scales HD351) will be measured following standard procedures, and an actigraph (the ActiGraph wGT3X-BT) will be provided to be worn on the child’s right hip 24-h a day for 1 week to measure usual sleep, physical activity and sedentary behaviour, initialized with 15 s epochs.

Families will be provided with a Participant Instruction Booklet containing all study instructions. After the baseline appointment is complete, participants will be randomised to start with either sleep restriction or sleep extension during intervention weeks 3 and 5. Randomisation will be stratified by age (grouped by 8–10 and 11–12 years) and gender. Randomisation to order will be generated using random block lengths (Stata 15.1, StataCorp, Texas) and uploaded into the REDCap randomisation module [67].

### Intervention

Data from three sources (questionnaire detailing usual sleep times, sleep diary records, and actigraphy) will be triangulated to determine usual sleep onset and offset for each child, separately for weekdays and weekends. The actigraphy results will be used to guide personalised intervention during experimental weeks 3 and 5 (e.g. double checking with caregivers that data gathered reflected usual behaviour). Based on their randomisation group, each child will be instructed to go to bed 1 hr later or earlier than their usual bed time each night for seven nights, while maintaining the usual wake up time determined during baseline week 1. Following a week of no intervention (week 4) the child will then undergo the opposite intervention in week 5.

Naps will not be permitted during the study. Field researchers will work with families and children to determine how best to ensure adherence for each family using strategies such as pre-planning, problem solving and positive reinforcement, based on their individual screening results [68]. During the two sleep intervention weeks, daily bed time text reminders will be sent to either the primary caregiver, the child, or both, depending on individual request, to encourage adherence. Text reminders will also be sent to remind participants when to wear the cameras, as described below. These reminders will be sent by an online platform or wireless software application. Upon completion of the project, all child participants will receive a $100 voucher, and primary caregivers a $50 voucher, to reimburse them for their time.

### Measurement of sleep and activity

We will use the same procedures as during baseline (full week of 24-h measurement). ActiLife software (version 9.0.0) will be used to initialise and download the actigraphy data, and an automated script developed in MATLAB® (MathWorks, Natick, MA, USA) will be used for analysis of sleep and activity patterns. The automated MATLAB® script uses a count-scaled algorithm to estimate sleep onset and offset for overnight sleep and awakenings (wake after sleep onset, WASO) [69] specific to each individual for each day [70]. Once these sleep data are effectively “removed” from the 24-h day, time spent sedentary and at various intensities of activity can be determined. Non-wear time will be defined as at least 20 min of consecutive zeros during awake time data [71]. The cut-offs of Evenson et al [72] will be used to denote time in sedentary, light, moderate and vigorous activity as recommended by Trost et al [73] for our age range. Appropriate statistical techniques will be used to determine whether the loss in sleep time decreased time spent being active or increases the proportion of time spent sedentary [48, 63].

### Main outcome assessments (weeks 3–5)

While lead researchers will need to know participant allocation in order to advise bed time changes, the study administrator, researchers collecting outcome data and those conducting statistical analyses will be blinded to participant allocation. Data collection will occur at baseline (week 1) and during the two experimental weeks (weeks 3 and 5) to test the two sleep conditions (restriction and extension). Some measurements occur daily during intervention weeks (actigraphy), on 2 days (diet recalls, cameras) or only at the end of each experimental week (hunger experiment, food cravings, questionnaires).

#### Clinic visit: eating in the absence of hunger (EAH) feeding experiment (primary aim)

**Fig. 2:**
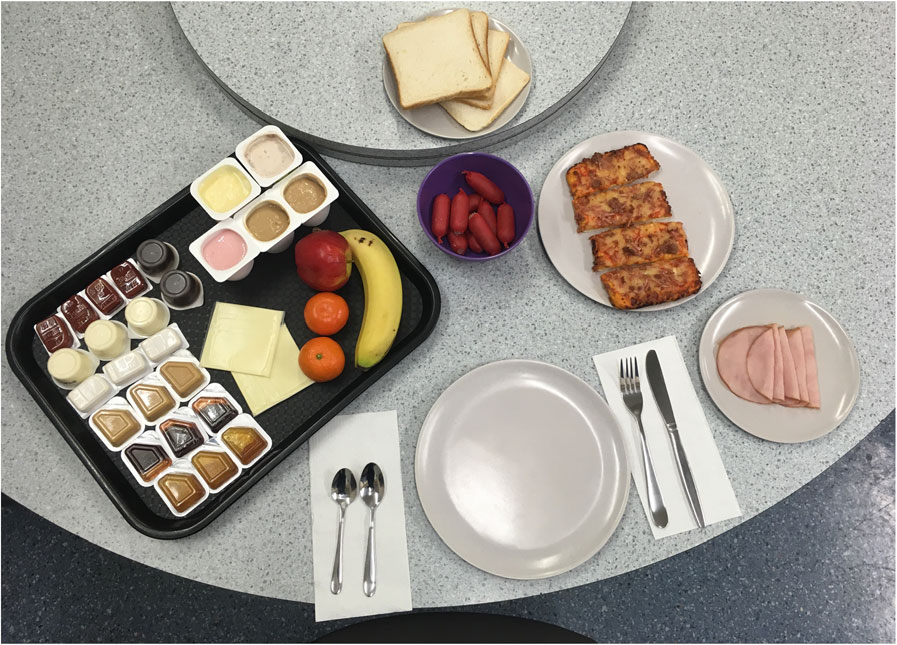
Photos of phase 1 (pre-load) meal set-up from the feeding experiment (photo courtesy of DRM and SM)

**Fig. 3:**
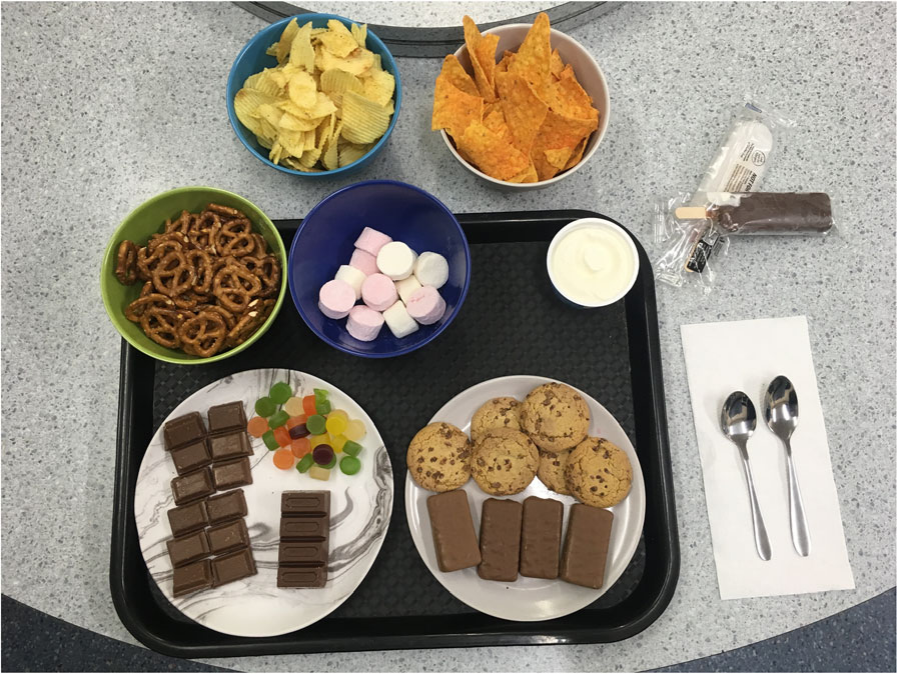
Photos of phase 2 (free access) meal set-up from the feeding experiment (photo courtesy of DRM and SM)

Our clinic-based feeding experiment in the late afternoon on the last day of each experimental week (weeks 3 and 5), will carefully measure eating in the absence of hunger objectively, by assessing first how participants report their preferences for certain foods via questionnaire, and then how participants consume treat foods under each sleep condition on a ‘full stomach’ [74]. Upon arrival at the clinic, caregivers and participants will complete a 24-h diet recall. The children will complete the Food Cravings Questionnaire and be weighed. They will then begin their pre-load meal which contains the pre-weighed and measured items listed in Table 3 and shown in Fig. 2**.** The pre-load meal will be offered as a buffet dinner, at which time they will eat ad-libitum until they are fully satisfied. When children appear to be replete, ongoing intake will be gently encouraged to assure children eat until they feel satisfied or ‘full’. The feeling of repletion will be assessed by a validated hunger and satiety visual 1–5 point rating scale called “Teddy the Bear” that is designed for primary school children [75]. Only those children who indicate fullness of 4 or 5 will be included in analyses to ensure children were full before the free access phase [76]. Once they are finished eating, the time will be noted and the child participant moved into a separate room for questionnaire completion. Based on other research, we piloted this protocol with a 15 min gap between the child finishing the pre-load meal, and beginning to eat in the free access phase [74], and we will continue this practice in the main study. Caregivers will complete their own questionnaires while the children complete the free access phase of the experiment, which contains the pre-weighed and measured items listed in Table 4 and Fig. 3, where participants will be given the opportunity to eat highly palatable snacks (e.g. lollies, chips, chocolate) for 15 min without adult supervision.

**Table 3:** Foods offered during the pre-load phase of the Eating in the Absence of Hunger (EAH) experiment

Food item	Quantity (g)	Energy content (kJ) [1]
Pizza	300	3036
Cocktail sausages	240	2124
Tomato sauce	60	243
Barbeque sauce	60	415
White bread	122	1214
Margarine	30	726
Garlic aioli	90	999
Sliced ham	50	206
Processed cheese	42	542
Jam	45	453
Honey	45	545
Peanut butter	33	856
Fruit yoghurt (low fat, sweetened)	300	986
Dairy food yoghurt (low fat, sweetened)	300	1020
Banana	200	764
Apple (Eve, red)	200	434
Mandarins	170	323
Total		14,883 [2]

^1^ All values obtained from manufacturer’s Nutrition Information Panels (NIPS) except for fruit, for which values are from Kaiculator software

^2^ Total is > 100% estimated energy requirement (EER) for boys and girls aged 8-12 years [65]

**Table 4:** Foods offered during the free access phase of the Eating in the Absence of Hunger (EAH) experiment

Food item	Quantity (g or ml)	Energy content (kJ)^1^
Potato crisps	90	2025
Corn chips	90	1953
Pretzels	100	1640
Cadbury diary milk chocolate	96	2160
Whittakers milk chocolate	50	1177
Chocolate chip biscuits	60	1116
Chocolate-covered malt biscuits	72	1570
Fruit-flavoured gummy lollies (wine gums’)	60	858
Marshmallows	100	1340
Chocolate-covered ice-cream on a stick with jelly	53 ml (43 g)	456
Vanilla ice-cream	100 ml (53 g)	458
Lemonade popsicle	53 mil (55 g)	163
Total		14,955 [2]

^1^ All values obtained from manufacturer’s Nutrition Information Panels (NIPS)

^2^ Total is > 100% estimated energy requirement (EER) for boys and girls aged 8–12 years [65]

Foods offered in both phases will be weighed both before and after the child eats, in order to calculate the amount of each food consumed, in grams. Energy content will be obtained from the Nutrition Information Panel (NIP) of each manufactured food product, or from the nutritional assessment software Kaiculator (University of Otago) programme if a NIP is not available. The amount of food consumed will be determined by weighing of foods and packaging offered in each phase before and after the child eats, and will then be used to calculate total energy intake for each child, in each phase to establish whether children consume more energy (kJ) when eating in the absence of hunger following a week of sleep restriction, compared to a week of sleep extension [10, 77]. The food preference questionnaire from baseline will be used to accommodate any intolerances and allergies (gluten, dairy, soy, any others) as well as any significant dislikes, and will be reviewed well in advance of the feeding experiment. In the case of intolerances or dislikes, foods of similar energy value will be provided and changes noted. Water will be made available upon request.

#### Diet recalls (secondary aims 1, 2 and 3)

Children (assisted by their caregivers) will be interviewed about the child’s dietary intake on the third day and the last day of each experimental week (weeks 3 and 5) using multiple pass 24-h recalls, the type of method used in the NZ Children’s National Nutrition Survey **[**65**].** In-person diet recalls will occur on the days directly after the cameras are worn (see below). Caregiver/child dyads will be interviewed in person about their child’s dietary intake (from midnight to midnight), on 2 days during each test week (ensuring all days of the week are represented across conditions). Collecting two 24-h recalls will the calculation of ‘usual intake’ using the Multiple Source Method for estimating usual dietary intake **[**78**].** This method takes into account day-to-day variation so that each participant’s intake estimate for that week will be more representative of the whole week, rather than being overly influenced by one extreme day. The first step will collect a quick list of all foods and beverages consumed the previous day in the 24-h midnight to midnight period. Next, information will be sought regarding the time and place of eating, which act as memory cues to help the participant remember the details about food in the next step. In step three, comprehensive details regarding each food or beverage item will be collected including brand name, cooking method, and portion size. A variety of aids will be used to assist with portion size estimation including photographs of commonly consumed foods, and using dried beans to estimate the volume of food consumed (this can be converted to grams of each item using known data on density where weight = volume x density). A list of typical forgotten foods will be presented (e.g. beverages, sweets, snacks), which can account for up to 6% of energy each day **[**79**].** Finally, the recall will be reviewed and additional foods probed **[**79**,** 80**].** The diet recalls will be recorded on paper and subsequently entered into FoodWorks 9 Professional® using the Otago Food list 2018 **[**81**],** and energy intake will be calculated and compared between extension and restriction interventions. Change in food group consumption will also be compared.

#### Wearable cameras (secondary aims 1, 2, 3)

Participants will wear a small camera (Brinno TLC130), set to automatically take a photograph every 2 seconds for 2 days during each test week; the cameras will be worn during all waking hours on the days prior to collecting the in-person 24-h diet recall interviews. Thus the camera images assess the same day as the recalls, as 24-h diet recalls assess the previous day’s intake. By timing the outcome measures in this way, the images can be reviewed in order to seek any clarifications to improve the accuracy of dietary recalls conducted by field researchers to elucidate food intake that appears in the camera images but not the diet recalls. Studies in adults have shown that the addition of these images reduces under-reporting of foods [82, 83], particularly snacks [37], although little work has been undertaken in children. The images will also be used to map the context behind eating on these days, providing insight into alterations in food choice in the free-living environment [83, 84]. Once images containing foods have been identified these images will be coded using the open source software TimeLapse2, which allows users to configure their own coding schemas and generate a database as images are coded. Images will be coded to determine information on food type (342 possible codes), location of consumption, length of eating occasion, presence of other people, and simultaneous participation in other activities [40]. Participants and their caregivers will have the option to view these photos prior to the researchers seeing them, and will have the opportunity to delete what they want, to maintain privacy. We have previously used this method to code screen images in teenagers [85]. Any images used for presentation purposes will require written consent from families, and any third party individuals faces will be blurred.

We will follow the ethical framework developed by Kelly et al [86] which guides researchers using automated wearable cameras and considers participant privacy, anonymity and storage of data. Participants and their families will be offered the opportunity to view and delete images before the research team views them. During the study participants will also be able to remove the camera or turn it off and on when they require privacy. Images will be stored on a secure server only accessible to researchers involved in the study. All data will be anonymised using participant ID. The camera may capture third-party members of the public including minors. Any identifying features within an image will be blurred before any image is used for publication or presentation purposes. Participants will carry an information card which they can give to members of the public explaining the study. They will also be instructed to simply remove the camera if asked to do so.

#### Questionnaires (end of each experimental week)

##### Changes in eating behaviour (secondary aim 3)

Primary caregivers will complete the 35-item Child Eating Behaviour Questionnaire (CEBQ) [64], which provides eight subscales including satiety responsiveness (how well they eat to appetite), food responsiveness (eating for reasons other than hunger), emotional over-eating (eating because of emotions), and fussiness. Questions will be adapted to reflect the past week.

##### Assessment of mood and sleep impairment/disturbance (secondary aim 3)

Children will complete the 27-item Kidscreen questionnaire which assesses mood and quality of life over the past week [61], and the 16-item PROMIS patient-reported outcomes measurement information system (PROMIS) questionnaire which assesses sleep disturbances [62] (difficulties falling and staying asleep) and sleep impairment [62] (impact on daytime functioning) over the past week. Caregivers will complete a proxy version of the PROMIS questionnaire (providing different perspective of the outcomes).

##### Food cravings questionnaire (secondary aim 4)

The food cravings questionnaire is a computerized task that measures the desire to eat a variety of foods based on an American model [32, 33] modified to represent New Zealand foods. Children will be shown pictures of a variety of foods (n = 60) differing in nutritional quality, including core and non-core foods. ‘Core’ foods form the five food groups (fruit, vegetables, cereals, meat and alternatives, and milk and alternatives), and ‘non-core’ foods are everything else, defined by fat and sugar cut-off points derived from dietary guidelines [87]. Participants will be asked to rate the appeal of each food on a sliding scale reflecting how much they want to eat that food right now (from “*Heaps!*” to “*Not at all*”). It is anticipated that the treat foods will be rated more highly after sleep restriction.

##### Changes in parenting (secondary aim 5)

Caregivers will also complete a questionnaire about their child’s snack and meal habits during that week; items include questions such as *“I gave my child a snack to keep the peace”* and *“I cooked a meal that my child was more likely to eat when they were tired”.* Five answer options range from *‘never’* to *‘all the time’.* The questionnaire was developed by the research team based on existing questionnaires assessing parenting and snacking [88, 89].

##### Screen time and sedentary behaviour (secondary aim 6)

A screen time survey and sedentary behaviour questionnaire, developed by adapting questions from existing surveys [90, 91], and including original questions, will also be administered to caregivers.

### Other outcome measures

#### Anthropometry

As discussed, child participants will be weighed and measured following standard procedures. Height (Wedderburn Portable Height Rod, WS-HRP) will be taken only at baseline. Weight (Tanita electronic scales HD351) will be measured at baseline and at the two clinic visits at the end of week 3 and 5. Height will be measured twice; if the two measures are not within 0.5 cm of each other, a third measurement will be conducted. Weight will be measured twice at each occasion, to the nearest 0.5 kg.

#### Exit interviews

Qualitative feedback on intervention experience, barriers and enablers of following intervention, child behaviour change, and impact on parenting and family, will be assessed by semi-structured interviews. A researcher not involved with previous data collection will contact each family to schedule a semi-structured interview of caregiver/child dyads within 1 month of completing the study. The aim of the interviews will be explanatory [92], in order to obtain a more complete understanding of the experiences of those taking part in research. The interviews will be conducted face-to-face within participants’ homes. The interviewer will have no previous interaction with the participants and be independent of the main research studies.

The interviews will be audio recorded, transcribed and field notes made after each interview. Questions will cover the following topics:

- Positive and negative experiences relating to the study in general
- Adherence enablers and barriers
- Child behaviour change (food choice, mood, etc.) during sleep intervention weeks
- Parenting behaviour changes during sleep intervention weeks
- How the intervention impacted on the family
- Future intension regarding anything learned
- General intervention feedback

The transcribed interviews will be intensively scrutinized to recognize and interpret themes [93, 94]. The thematic analyses will take an inductive approach and will include familiarisation with the transcript, initial open coding, collating codes into themes or sub-themes, reviewing the themes and naming the themes [95, 96]. Identified themes will be independently reviewed by at least one other researcher, and then discussed. The qualitative software NVivo will be used to manage, store and support the data analysis.

#### Feasibility and pilot work

A full DREAM pilot was undertaken in 11 children aged 8–12 years to determine acceptability and feasibility (University of Otago Human Ethics Committee reference #18/044). All but one child completed all measurements; the child with incomplete data was sick for several days during week 5. Analyses of completed actigraphy and sleep diaries demonstrated that children were 100% adherent to the protocols and mean time in bed was 1.5 h different between sleep restriction and extension conditions. Importantly, sleep was reduced by almost one hour per night during the restriction week, proving it is feasible to reduce sleep in this age group. The adherence findings in the DREAM pilot study are in line with others who also report high compliance (95%) with a similar protocol in children of this age [56]. In terms of our primary outcome, only two children had to be excluded from analyses as they did not indicate scores of 4 or 5 (indicating they were full) on the Teddy Bear scale at the end of the preload phase of the EAH feeding experiment. One child was unable (due to school regulations) to wear their camera for all hours during the prescribed days; this resulted in an overall 91% adherence to camera protocols.

Feedback from participants was overwhelmingly positive. Children loved being involved and “*felt very grown up recording their bed times”.* Several commented how they *“felt much better during the sleep extension week”* with caregivers commenting they noticed differences in their child’s behaviour that were enlightening as many had not really considered how sleep might influence behaviour or food intake. Overall caregivers felt the study burden was manageable, even though the sleep restriction week was difficult for some families.

#### Power calculations and statistical analyses

Recruiting 110 children will allow for a representative sample, with 20% drop-out and incomplete data. For the objective experiment for our main outcome (eating in the absence of hunger at the feeding experiment), based on a standard deviation of 870 kJ and a within-person correlation of 0.7 [97], a sample size of 59 would be required to detect a difference of 250 kJ in energy intake between the two different sleep conditions (80% power, p < 0.05). Fifty-five children would be required to detect a difference in the eating in the absence of hunger subscales [98] of 0.3 standard deviation. These numbers will also allow us to detect important differences in energy intake (secondary outcome) from the diet recalls, over the total day (500 kJ difference, n = 85) or just at night (200 kJ difference, n = 50) [56]. Mixed effects regression models will be used to determine mean differences between the two conditions with participant identification number as a random effect which accounts for both within-person and between-person variation. Skewed data will be log-transformed as appropriate.

## Discussion

Diet and physical activity have long been the cornerstones of obesity research. It has been shown that toddlers with high body mass index (BMI) continue to have high BMI in adolescence [99], and that longer sleep during infancy and toddlerhood seems to be protective of normal BMI in early childhood [100]. However, it is unknown *why* shorter sleep might influence the development of obesity in children or how sleep restriction affects both child and family behaviours. We need to understand the underlying mechanisms of this relationship to inform the development of optimal intervention strategies.

## Abbreviations

BMI: Body mass index
CEBQ: Child eating behaviour
DREAM: Daily Rest, Eating, & Activity Monitoring randomized trial EAH Eating in the absence of hunger
g: grams
kJ: kilojoules
ml: millilitre
NIP: Nutrition information panel
NZ: New Zealand
PROMIS: Patient-reported outcomes measurement information system
REDCap: Research Electronic Data Capture
SDSC: Sleep disturbance scale for children
